# Adult cardiac surgery in Trinidad and Tobago during the COVID‐19 pandemic: Lessons from a developing country

**DOI:** 10.1111/jocs.14975

**Published:** 2020-08-26

**Authors:** Richard A. E. Ramsingh, Jean‐Luc Duval, Natasha C. Rahaman, Risshi D. Rampersad, Gianni D. Angelini, Giovanni Teodori

**Affiliations:** ^1^ Division of Cardiac Surgery, Caribbean Heart Care Medcorp St. Clair Medical Centre Port of Spain Trinidad and Tobago; ^2^ Department of Cardiology, Caribbean Heart Care Medcorp St. Clair Medical Centre Port of Spain Trinidad and Tobago; ^3^ Medical School King's College London School of Medicine London UK; ^4^ Department of Cardiac Surgery, Bristol Heart Institute University of Bristol Bristol UK

**Keywords:** adult cardiac surgery, COVID‐19, developing country, Trinidad & Tobago

## Abstract

**Background and Aim:**

The coronavirus disease 2019 (COVID‐19) pandemic has seen the cancellation of elective cardiac surgeries worldwide. Here we report the experience of a cardiac surgery unit in a developing country in response to the COVID‐19 crisis.

**Methods:**

From 6th April to 12th June 2020, 58 patients underwent urgent or emergency cardiac surgery. Data was reviewed from a prospectively entered unit‐maintained cardiac surgery database. To ensure safe delivery of care to patients, a series of strict measures were implemented which included: a parallel healthcare system maintaining a COVID‐19 cold site, social isolation of patients for one to 2 weeks before surgery, polymerase chain reaction testing for COVID‐19, 72 hours before surgery, discrete staff assigned only to cardiac surgical cases socially isolated for 2 weeks as necessary.

**Results:**

The mean age at surgery was 59.7 ± 11 years and 41 (70.7%) were male. Fifty‐two patients were hypertensive (90%), and 32 were diabetic (55.2%). There were three emergency type A aortic dissections. Forty‐seven patients underwent coronary artery bypass graft surgery with all but three performed off‐pump. Fourteen cases required blood product transfusion. One patient had postoperative pneumonia associated with chronic obstructive pulmonary disease. The median length of stay was 5.7 ± 1.8 days. All patients were discharged home after rehabilitation. There were no cases of COVID‐19 infection among healthcare workers during the study period.

**Conclusion:**

These strategies allowed us to maintain a service for urgent and emergency procedures and may prove useful for larger countries when there is decrease in COVID‐19 cases and planning for the restart of elective cardiac surgery.

## 1 INTRODUCTION

The coronavirus disease (COVID‐19), since December 2019, has had vast and far reaching effects in over 190 countries around the world.^1^ As of 17th June 2020, the World Health Organization (WHO) reports approximately eight million confirmed cases with 440,000 deaths worldwide.^2^ Since the importation of the first COVID‐19 case in Trinidad and Tobago on 12th March 2020, aggressive policy measures have been enacted in line with the WHO strategic objectives.^3^ The greatest impetus for this came with the exponential rise in COVID‐19 cases in March 2020 that prompted closure of national borders, nationwide lockdown, and suspension of all elective surgeries. With this emergent crisis, brought great uncertainty in healthcare; resources including personal protective equipment (PPE), ventilators, and testing became heavily regulated, new healthcare policies were enacted to ensure safety and reduced transmission, the impacts of which were unforeseeable. Nevertheless, identifying the most vulnerable patients with low probability of survival remained of prime importance.

The Ministry of Health of Trinidad and Tobago, under The Public Health (2019 novel coronavirus) Regulations, 2020, created a parallel system by designating isolation and quarantine hospitals for the sole purpose of the treatment and care of suspected and confirmed cases.^4^ This has resulted in unique conditions for the continuation of urgent and emergency surgery despite the cancellation of an estimated 28 million elective surgeries worldwide at the peak of the pandemic.^5^

We sought to explore the Trinidadian perspective during this pandemic and assess its impact on a cardiac surgery service after the implementation of the country's COVID‐19 regulations. It is essential to reflect on the effects of the pandemic foremostly to validate and inform practice in the likely event of a second wave, but also to contribute to the global understanding of response to this disease. As such, the goals of this communication are to report on the conditions that allowed continuation of emergency and urgent elective cardiac surgical cases during the COVID‐19 pandemic.

## 2 MATERIALS AND METHODS

From 6th April to 12th June 2020, 58 patients underwent cardiac surgery in two hospitals, St. Clair Medical Center, a private hospital, and Eric Williams Medical Sciences Complex (EWMSC), a public hospital. The cardiac surgical service at both hospitals is covered by the same surgical team. The study was performed as part of an approved audit, according to the specifications of the Declaration of Helsinki. We reviewed prospectively entered data from the unit maintained cardiac surgery database of consecutive patients.

During the reported COVID‐19 period we did not operate on elective cases. A patient was defined as elective if they do not require urgent surgery and has been placed on a waiting list (before COVID‐19 the waiting list was around 2‐3 months). The number of procedures performed before COVID‐19 was approximately 35 cases per month.

All patients were reviewed in our preoperative clinic with enforced social distancing in waiting rooms and mandatory mask wearing. Cases were screened by a multidisciplinary cardiac surgical team. Patients were selected on the basis of requiring urgent intervention (unstable angina, symptomatic aortic stenosis, poor ejection fraction) or emergency (acute type A aortic dissection).

Urgent patients identified according to the above criteria by the multidisciplinary cardiac surgical team were asked to socially isolate themselves at home before scheduled surgery for at least 7 to 14 days. Because of patient bed limitation, it was not possible to keep those patients in hospital for the required period of isolation. At St. Clair Medical Center, patients had a 72 hours preoperative COVID‐19 polymerase chain reaction (PCR) test. In the public hospital, EWMSC, cases were tested only if symptomatic (because of limited availability of reverse transcription‐PCR [RT‐PCR] testing) in concordance with the Ministry of Health's management of the ongoing crisis. The three emergency dissections were operated in the private hospital and tested for RT‐PCR.

All patients were intubated with minimum staff in the operating theater including an anaesthetist and an assistant with appropriate PPE (N‐95/visor) to protect the team and diminish the chance of in‐hospital “community spread.” The surgical team wore standard surgical masks, gowns, and protective loupes. All cardiac surgical patients were managed in an intensive care unit (ICU) and high dependency unit (HDU) (not exposed to noncardiac surgery patients) by staff dedicated to their care only.

## 3 RESULTS

The demographic and clinical data are summarized in Table 1. Urgent interventions were conducted in 55 patients, while three required emergency treatment.

**Table 1:** Patient characteristics

	(n = 58)
Age, y	59.7 ± 11.0
Sex	
Male	41 (70.7)
Female	17 (29.3)
Diabetes	32 (55.2)
Hypertension	52 (90)
Classification of intervention	
Urgent	55 (94.8)
Emergent	3 (5.2)
Indication for surgery	
Acute aortic dissection	3 (5.2)
Unstable angina	40 (69)
Symptomatic aortic stenosis	4 (6.9)
Reduced ejection fraction + stable angina	11 (19)

*Note*: Values are means ± SD, n (%).

The mean age at surgery was 59.7 ± 11 years and 41 (70.7%) were male. Fifty‐two patients were hypertensive (90%), and 32 were diabetic (55.2%). There were three emergency type A aortic dissections. Forty‐seven patients underwent coronary artery bypass graft surgery with all but three performed off‐pump. Fourteen cases required blood product transfusion. One patient had postoperative pneumonia associated with chronic obstructive pulmonary disease. The median length of stay was 5.7 ± 1.8 days (Table 2). All patients were discharged home after rehabilitation. There were no cases of COVID‐19 infection among healthcare workers during this period. There were no reported COVID‐19 positive patients admitted to either hospital during the study period.

**Table 2:** Surgical details

	(n = 58)
Operation	
Off‐pump CABG	47 (81)
Replacement of ascending aorta	3 (5.2)
On‐pump CABG + LV aneurysmectomy	2 (3.4)
On‐pump CABG	1 (1.7)
AVR + CABG	3 (5.2)
AVR	2 (3.4)
Complications	
Pneumonia	1 (1.7)
Re‐opening for bleeding	0
Stroke	0
Renal failure requiring dialysis	0
Length of hospital stay, d	5.7 ± 1.8
Discharge home	58/58 (100)

*Note*: Values are means ± SD, n (%).

Abbreviations: AVR, aortic valve replacement; CABG, coronary artery bypass graft surgery; LV, left ventricle.

## 4 DISCUSSION

Healthcare service provision around the world has been unprecedentedly altered because of the COVID‐19 pandemic. Currently, the literature pertaining to the effects on cardiac surgical services comes exclusively from large countries that have experienced widespread viral spread and subsequent overwhelming of health care systems, particularly critical care facilities. Reports from countries, such as the United Kingdom, Italy, and Canada have highlighted the following strategies in dealing with the pandemic: (a) complete cessation of elective surgery with centralization of emergent and urgent services to a few dedicated centers, (b) meticulous multidisciplinary triage of patients with divergence to interventional services where appropriate due to associated shorter length of stay, (c) redeploying of cardiac surgical staff, equipment and beds to expand critical care services, and (d) largescale uptake of virtual and tele‐medicine.^6^, ^7^, ^8^, ^9^ These measures are appropriate to the scale of the pandemic in their respective countries and may provide a useful template, but do not necessarily represent a universal gold‐standard response. Many countries, Trinidad and Tobago included, may have experienced a much lower COVID‐19 burden and as such may require a more tailored approach to service provision during the pandemic.

The population of our twin‐island state is small relative to many of the worst hit countries worldwide, standing at a modest 1.35 million.^10^ At the time of writing in June, a total of 4152 tests have been performed, with only 123 positive results identified and eight COVID‐19‐associated deaths.^11^ Under the Public Health Regulations, the Ministry of Health of Trinidad and Tobago designated specific facilities with the sole purpose of isolating and treating suspected and confirmed cases of COVID‐19.^4^ These centers were manned by a dedicated parallel work force who were isolated themselves to protect their families and the broader community. These policy reforms were further supported by contingency changes within the public healthcare sector. Remarkably, a downstream effect was observed, as cross‐contamination was prevented and other hospitals, including EWMSC and St. Clair Medical Center, were able to remain COVID‐19 cold to date.^12^ Furthermore, in the public healthcare sector, initially a decision was made to cancel elective surgery to conserve PPE (gloves, masks, and gowns) and ring fence ward and ICU beds to accommodate potential overflow from designated COVID‐19 facilities. However, despite the pandemic and the lockdown, there remains a duty of care to ensure vulnerable cardiac patients access the intervention they require, otherwise there may have been an increase in cardiac mortality.

In the period examined, 58 cases deemed urgent or emergency were performed, with no hospital mortality. To optimize outcomes and protect both patients and staff in these instances, consideration of patients' COVID‐19 status was viewed as paramount. All cardiac surgery patients at the private center, were required to self‐isolate for one to 2 weeks and underwent PCR testing 72 hours before surgery. Isolation was also implemented for patients in the public hospital. However, we were unable to routinely screen patients for PCR in the public hospital, classified as a COVID‐19 cold site, given the limited availability of testing nationally. It is important to note that, postoperative cardiac surgical patients in the ICU require intense chest physiotherapy and nebulizing for the purposes of coughing and expectorating. As both procedures are aerosolizing coupled with inapplicability of surgical masks, asymptomatic patients were duly screened with PCR when possible not only for the safety of other patients, but for healthcare workers as well. Similar protective strategies were also employed in the postoperative period, as cardiac patients were batched and cared for in one designated ICU/HDU. Visitors were also limited. The net effect of these precautionary steps facilitated individual patient safety, contributed to maintaining the integrity of parallel, discrete healthcare systems, and created a setting that allowed for the unencumbered continuation of cardiac surgery throughout the pandemic.

While our smaller population confers these advantages, there are also associated drawbacks. The period of lockdown saw an absolute decrease in blood donations, which in the context of a smaller population created a national shortage of blood products. Notably, the routine practice of our cardiac surgical team is off‐pump coronary surgery which is associated with a decreased rate of blood transfusions compared with on‐pump procedures.^13^, ^14^ Fortunately, further deficit in blood products were able to be overcome with the use of cell saver machines. Additionally, though a smaller population of cardiac surgery patients, clinics continued to be carried out face‐to‐face with social distancing and use of masks, and therefore did not see the uptake of telemedicine seen in larger countries.

We recognize that these strategies proven viable in Trinidad cannot necessarily be generalized regardless of population size or infections rates, as many other confounding factors exist between countries and healthcare systems. Despite this however, several lessons may be derived from our unique situation, which may prove useful in guiding our neighbors' own unique circumstances. To ensure safe delivery of care to patients, a series of strict measures were implemented which included: a parallel healthcare system maintaining a COVID‐19 cold site, social isolation of patients for one or 2 weeks before surgery, whenever possible PCR testing for COVID‐19, 72 hours before surgery, discrete staff assigned only to cardiac surgical cases socially isolated for 2 weeks as necessary. Screening of patients should be an essential component of any major elective cardiac surgical service that plans to continue throughout the pandemic or in the event of a second wave. These strategies allowed us to maintain a service for urgent and emergency procedures and may prove useful for larger countries when there is decrease in COVID cases and planning for the restart of elective cardiac surgery.

## 5 CONCLUSION

Urgent and emergency cardiac surgery can be safely performed during the COVID‐19 pandemic provided that certain precautions are taken. This experience confidently yet cautiously prepares us if there is a second wave of COVID‐19 infections worldwide.
